# Biomarkers in spinal muscular atrophy

**DOI:** 10.3389/fneur.2025.1636992

**Published:** 2025-07-18

**Authors:** Liping Yan, Jinping Zhang, Jian Zheng, Hua Hao

**Affiliations:** ^1^Department of Pathology, Second Affiliated Hospital of Nanchang University, Nanchang, China; ^2^Department of Neurology, Affiliated Hospital of Shandong University of Traditional Chinese Medicine, Jinan, China; ^3^Department of Pathology, Yangpu Hospital, School of Medicine, Tongji University, Shanghai, China

**Keywords:** SMA, SMN1, molecular biomarkers, physiological biomarkers, imaging biomarkers

## Abstract

Spinal muscular atrophy is a hereditary disorder leading to severe neuromuscular impairment. With the introduction of disease-modifying therapies in recent years, the role of biomarkers has expanded from aiding diagnosis to monitoring treatment responses, prognostic assessment, and the development of individualized treatment strategies. This review systematically summarizes biomarkers in the field of spinal muscular atrophy, including physiological indicators, functional assessments, imaging features, and molecular markers, which are derived from the analysis of different tissues from human patients and animal models. This article provides a concise summary of the classic biomarkers widely used in current clinical practice and introduces the potential new biomarkers revealed by the latest research. It focuses on discussing the expression patterns, clinical correlations, and applicable conditions of various types of biomarkers, with the aim of providing more accurate basis for disease stratification, efficacy prediction, and treatment decision-making.

## 1 Introduction

Classic spinal muscular atrophy (SMA) is an autosomal recessive neuromuscular disease characterized by homozygous deletion of the survival of motor neuron 1 (SMN1) gene on chromosome 5q13.2 (1, 2). SMA affects populations worldwide, and its carrier frequency, incidence, and prevalence vary across regions, ethnicities, and genders. It has been reported that Caucasian and Asian populations carry a higher frequency of SMN1 mutations than populations of sub-Saharan African ancestry. However, carrier frequency cannot be directly translated to incidence and prevalence because the frequencies of very severe (intrauterine lethal) and very mild (asymptomatic in adults) phenotypes with biallelic SMN1 mutations are unknown (3). The analysis of pooled data based on TREAT-NMD Global SMA Registry and Cure SMA member database showed that the proportion of male SMA patients is higher than that of female patients, and the clinical symptoms of male patients are more significant, and the proportion of male family members is also higher. Meanwhile, head circumference data suggest that male patients may have more significant brain developmental abnormalities (4). Pure deletions of exons 7 and 8 or a single exon 7 of the SMN1 gene are present in approximately 96% of cases, but there is no direct correlation between the types of SMN1 genetic defects and the severity of the clinical phenotype; the remaining patients showed SMN1 point mutation and SMN1 deletion, or extremely rare biallelic point mutation (5, 6).

Both SMN1 and SMN2 are located in the 5q13 region of human chromosome 5. Both have 99% sequence homology; however, the c.840C > T variant in exon 7 of SMN2 results in aberrant splicing of SMN2 and less than 10% efficiency of functional full-length SMN protein production (7). Notably, the incidence of pure deletions of the SMN2 gene is approximately 3–5% in the general population, but does not increase the incidence of clinical phenotypes due to normal SMN1 expression (8). In patients with SMA types 2–4, gene conversion of SMN1 to SMN2 results in an increased number of SMN2 copies (9). Reverse gene conversion can integrate SMN2 sequences into the SMN1 locus, resulting in an increased number of SMN1 copies; however, this can result in an increased risk of delayed-onset motor neuron disease [e.g., amyotrophic lateral sclerosis (ALS)], suggesting that excessive SMN expression can be harmful to motor neurons (10).

Werdnig and Hoffmann first reported cases of SMA type 1, which they named Werdnig–Hoffmann disease. In 1956, Kugelberg and Welander reported Kugelberg–Welander disease, now known as SMA type 3 (11). Three common subtypes of classic SMA have been defined based on onset age and motor ability of untreated patients (7). SMA type 1 is the most common type, with an onset within the first 6 months of life, and death usually occurs within 2 years of age due to respiratory failure (mortality rate > 90%). Patients with SMA types 2 or 3 have relatively mild symptoms. The onset of SMA type 2 occurs at 6–18 months of age, and patients can sit unaided but are unable to walk. Patients with SMA type 3 can walk independently but gradually lose motor ability. Two other, rare subtypes of classic SMA have also been described. Type 0 has a prenatal disease onset; patients may present with reduced or absent fetal movements in utero, asphyxia, and severe weakness at birth, and they often survive only for days to weeks (12). Type 4 is characterized by adult onset; patients with this subtype have the mildest symptoms and a survival rate close to normal (13). Studies have shown that 80% of patients with SMA type 1 have one or two SMN2 copies, 78% of patients with SMA type 2 have three SMN2 copies, and 93% of patients with SMA type 3 have three or four SMN2 copies (7).

The diagnostic process for SMA can be divided into three key stages: preconception carrier screening, prenatal testing of the fetus, and postnatal testing of the neonate or infant. This tiered diagnostic system is important for early intervention and improving patient prognosis. Preconception carrier screening can help couples make informed reproductive decisions (14). Postnatal SMN proteins in the spinal cord are age-dependently depleted, and delayed treatment will accelerate the pathology (15). Early identification of patients with SMA in the presymptomatic stage and implementation of interventions considerably improve the prognosis of these patients (16), contribute to the improvement of their quality of life, and reduce the burden on society. Genetic testing is the gold standard for screening SMA carriers and diagnosing patients affected by SMA.

Multiplex ligation-dependent probe amplification (MLPA) was once considered the method of choice for the molecular detection of SMA (2), but digital polymerase chain reaction (dPCR) has recently been shown to exhibit better accuracy in detecting copy number variants (17). In addition, the integration of long-read sequencing technology with conventional next-generation sequencing assays can improve the resolution of structural variants and avoid false-positive or false-negative gene detection (18).

With the application of disease-modifying therapies (e.g., nusinersen, onasemnogene abeparvovec, and risdiplam), the survival and motor function of most patients with SMA have improved. However, the therapeutic efficacy in some treated patients is low (19). New biomarkers, especially molecular ones, have been discovered in recent years through studies of amniotic fluid/chorionic villus samples, blood samples, cerebrospinal fluid (CSF), and skeletal muscle tissues of patients with SMA. Understanding the characteristics of these markers not only improves the sensitivity of carrier screening and presymptomatic diagnosis but also dynamically monitors the progression of the disease, thus guiding clinical work.

## 2 Molecular biomarkers

### 2.1 SMN protein levels

The expression level of SMN proteins is significantly correlated with the clinical type of SMA (types 1–4) and the copy number of the SMN2 gene (20); the higher the copy number of SMN2, the higher the expression of functional SMN proteins and the lower disease severity. SMN is widely expressed in various tissues and cell types, but the level of SMN expression is higher in the spinal cord (21). The spinal cord is the target organ for SMA therapy, but the difficulty in sampling spinal cord tissues limits its use in related studies. Typically, tissues available for biomarker studies are the blood and CSF. SMN is localized in the cytoplasm, nucleus (Cajal vesicles and gems), and nucleolus (22). SMN is involved in small nuclear ribonucleoprotein (snRNP) assembly and mRNA transport in the cytoplasm. SMN was enriched in bodies of Cajal (CBs) and gems, and CBs was the site of snRNP maturation and modification. SMN can be localized to the nucleolus in neurons and under stress conditions and may be related to ribosomal RNA processing or stress response. One imaging study quantified SMN protein expression levels in peripheral blood mononuclear cells using flow cytometry to obtain SMN spot values. In this study, SMN protein levels in maternal peripheral blood were positively correlated with those in neonatal umbilical cord blood (15). This suggests that it is possible to analyze maternal peripheral blood to predict the severity of postnatal SMA in the child and that induction of maternal SMN expression might be an effective therapeutic strategy for the fetus. The latest study to practice and report this treatment. Fetal type 1 SMA was confirmed by the investigators through family history and amniocentesis. Subsequently, Risdiplam was administered orally to the mother in the third trimester, and treatment was started on day 8 of life. So far, the child has not developed clinical symptoms of SMA (23). In another study, real-time counting of primary fibroblasts based on skin biopsy-derived primary fibroblasts efficiently quantified the amount of SMN protein-rich gems in the nucleus, and these values were highly positively correlated with the functional recovery of SMN proteins and the level of full-length SMN transcripts (24).

Both the amount of SMN protein and the number of gems, which are considered biomarkers of SMA, can be used to monitor disease progression and therapeutic efficacy. However, it remains unclear whether peripheral blood and central nervous system (CNS) SMN levels are directly correlated, with only a few reports confirming that increased peripheral blood SMN levels may reflect SMN levels in the CNS (25). The high cost of SMN protein assays may prevent their large-scale clinical dissemination. Manual microscopic counting of the number of gems in the nucleus is simple and easy to perform; however, subjective judgment errors may occur, and uniform cutoff values have not been established yet.

### 2.2 Biomarkers associated with synaptic dysfunction

The clinical manifestations of skeletal muscle weakness in patients with SMA are caused by the destruction of SMA motor units. Studies on human necropsy tissues and mouse models have shown that the initial stages of the disease are characterized by developmental disorders of the neuromuscular system including an abnormal acquisition of excitatory synaptic inputs to motor neurons, disturbed firing patterns in mature motor neurons, impaired radial growth of motor axons and Schwann cell sheaths, and reduced myelin formation. Impaired refinement of the synaptic structure, increased quantum content at the neuromuscular junction, and abnormal muscle fiber growth have also been reported (7). Recent studies have identified dysfunction and loss of proprioceptive synapses as key features of SMA pathology by analyzing patient specimens and mouse models of SMA (26). These findings strongly suggest that synaptic dysfunction plays a key role in the pathogenesis of SMA.

Hsc70-4 and HspA8 are members of the heat shock protein 70 family. These homologs of the same protein in different species play key roles in preventing protein aggregation and assist in the repair and degradation of misfolded proteins (27). In a mouse model, the G470R mutation in HspA8 significantly alleviated the severity of SMA by stabilizing the interaction between HspA8 and chaperone proteins, promoting the assembly of the SNARE complex at the neuromuscular junction, and directly enhancing synaptic function (28). In the Drosophila model, Hsc70-4 was identified as a chaperone protein that preferentially binds to SMN mutants, and the TDP-43 mutation triggers the downregulation of Hsc70-4/HspA8, which in turn disrupts synaptic function (29).

Plastin 3 (PLS3), identified as the first protective modifier in SMA, plays a key role in endocytosis, synaptic function, and cytoskeletal remodeling (30). PLS3 is an X-linked gene, and all asymptomatic SMN1-deficient individuals who exhibit PLS3 upregulation are female. These women have a 40-fold higher level of PLS3 expression compared to their diseased siblings, whereas men generally show reduced levels of PLS3 (31). Calcineurin-like EF-hand protein 1 (CHP1) is a directly interacting protein of PLS3 (32). In various SMA models, decreases in CHP1 expression promote calmodulin phosphatase activity and improve impaired synaptic endocytosis.

Thrombospondins 4 (TSP4), a member of the thrombospondins family, is mainly expressed in the heart and skeletal muscle in adult tissues and accumulates in the neuromuscular junction (NMJ), a specialized synaptic structure, and certain synapse-rich structures (33, 34). In SMA, in addition to the lack of SMN protein, retrograde signaling from the NMJ and skeletal muscle may also contribute to the damage of alpha motor neurons (α-mns) and significantly affect the overall clinical manifestations of the disease (35). Recent studies have found that the changes of TSP4 in the cerebrospinal fluid (CSF) of SMA patients are age- and disease-specific: its level is significantly reduced only in children with SMA, but not in adults. Meanwhile, this reduction is specific to SMA, and similar changes have not been observed in the CSF of patients with other pediatric neurological diseases (e.g., multiple sclerosis, peripheral neuropathy, meningitis/encephalitis) (36). More importantly, TSP4 levels in CSF increased in pediatric SMA patients after the first dose of Nusinersen, suggesting that TSP4 has the potential to be a biomarker for monitoring treatment response in pediatric SMA patients. However, further studies are needed to clarify its specific mechanism of action and clinical application value.

Abnormalities in synaptic and molecular chaperone functions may precede the onset of clinical symptoms. Changes in the expression or activity of proteins associated with synaptic function may reflect disease severity and help in the early detection of disease and the assessment of therapeutic responses (37). Therefore, targeting synaptic dysfunction may be a future strategy for adjuvant therapy. However, synaptic dysfunction and protein disorders involve multiple molecules, and a single biomarker may not be sufficient to fully reflect the disease state. Analyses of synaptic vesicle cycling and molecular chaperone function require highly sensitive assays, which may be difficult to generalize for routine clinical applications. Currently, relevant studies are mainly conducted in animal and cell culture models, and further clinical studies are required to verify the reliability of these assays.

### 2.3 Non-coding RNAs

Non-coding RNAs (ncRNAs) can be detected in various ways. ncRNAs, RNA molecules that do not encode proteins, are involved in cell proliferation, differentiation, metabolism, stress response, and other cellular processes through the regulation of gene expression. At present, the research focus is mainly on regulatory ncRNA. According to their length and structure, regulatory ncRNAs are divided into short ncRNA, long ncRNA (lncRNA), and circular RNA (circRNA).

MiRNA belongs to short chain ncRNA, and its expression level is closely related to muscle atrophy caused by various causes (38, 39). In the field of SMA research, increasing evidence has revealed characteristic aberrant miRNA expression profiles (see Table 1 for a summary of the characteristics and applications of some representative mirnas). Among them, muscle-specific miRNA (myomiR) is relatively well studied: miR-133a and miR-133b mainly regulate and promote the proliferation of myoblasts, while miR-206 and miR-1 are mainly involved in the regulation of myoblast differentiation. In addition, miRNA expression changes in motor neurons and glial cells have received much attention. Combined detection of miRNA and neurofilament proteins, such as pNF-H, may provide a more comprehensive assessment of therapeutic effect. It should be noted that the expression of some mirnas is tissue specific, while neurofilament proteins also have some limitations as biomarkers. Therefore, the influence of these factors on the results must be fully considered when designing and applying the combined detection strategy of miRNA and neurofilament protein.

**Table 1:** Characteristics and application analysis of SMA-related mirnas.

miRNA	Sample source	Specificity of tissue	Expressed characteristics	Clinical correlation	Application context	Reference
miR-206	Human peripheral blood	Skeletal muscle; Myocardium; Spinal motoneurons	100% of SMA patients have extremely low expression.	To distinguish SMA from DMD.	Diagnosis; Potential therapeutic targets	(99)
miR-208 (miR-208a)	Human peripheral blood	Skeletal muscle; Myocardium	Low expression in 75% of SMA patients.	It reflects the state of the myocardium and indicates the presence of cardiomyopathy.	Differential diagnosis	(99)
miR-191	Human peripheral blood	Unknown	100% of SMA patients have low expression.	Low expression is associated with increased neurodegeneration of SMA.	Combined with miR-206 for diagnosis	(99)
miR-206; miR-133a; miR-133b; miR-1	Skeletal muscle in the mouse model; Human peripheral blood	Skeletal muscle	During the onset stage, miR-206, miR-133a and miR-1 were significantly upregulated, while miR-133b showed an upregulation trend but not significantly.	Decreased miRNA expression after treatment * was associated with improved function; Markers of muscle atrophy.	Diagnosis; Prediction of treatment response; Monitor disease progression; Potential therapeutic targets	(100)
miR-206; miR-133a; miR-133b; miR-1	Human peripheral blood; Literature review	Skeletal muscle	There was a significant decrease after treatment *.	The decrease of miR-133a ≥ 6.6 Ct value can predict the improvement of HFMSE ≥3 points; Markers of muscle atrophy.	Prediction of treatment response; Monitor disease progression; Potential therapeutic targets	(101)
miR-206; miR-133a-3p; miR-133b; miR-103b; miR-1-3p	Cerebrospinal Fluid	Skeletal muscle	The level of miR-206 in non-responders was 1.8 times higher than that in responders. Respondents miR-103-b 2.6 times higher than those without reply; The expression of miR-1-3p, miR-133a-3p and miR-133b was lower in the responders.	MiR-206 baseline and negatively correlated with HFMSE improve miR-133-a-3 p and miR-206 joint to improve prediction accuracy.	Prediction of treatment response; Monitor disease progression; Potential therapeutic targets; To help identify treatment * responders	(102)
miR-146a	SMA astrocytes derived from IPscs in SMA patient; Spinal cord tissue of a mouse model; Cerebrospinal Fluid	Astrocyte	The only highly and significantly upregulated miRNA in SMA astrocytes (including after treatment *).	Markers of disease progression, neuroprotective targets, and regulation of neuroinflammation.	Diagnosis (in combination with other inflammatory markers to improve diagnostic specificity); Monitor disease progression; Potential therapeutic targets	(71, 103)
miR-132; miR-218; miR-9	Cerebrospinal Fluid; The spinal cord of a mouse model	Unknown	miR-132 and miR-9 were generally down-regulated in animal models. Expression was increased in CSF after treatment *. MiR-218 cut in animal models; The expression showed an upward trend after treatment.	Increased expression of miR-132, miR-218, and miR-9 was associated with improved motor function.	Treatment response monitoring	(103)
miR-34	Mouse model; Cerebrospinal Fluid	Spinal motoneurons	The expression of miR-34 family members (miR-34a/b/c) was significantly down-regulated, and miR-34 expression levels were further decreased after treatment*	The baseline level of miR-34b can predict the improvement of motor function in patients after 1 year (predicting ability ≧pNfH). MiR-34 expression changes associated with HINE – 2 score improved significantly. MiR-34 NMJ missing lead to damage.	Diagnosis; Prognosis; Monitoring of treatment response; Potential therapeutic targets	(104)
miR-34a (miR-34a-5p and miR-34a-3p)	Mouse model	Spinal motoneurons; Myocardium; Hepatocyte	In the pathogenesis stage, miR-34a-3p was first up-regulated and then down-regulated in the spinal cord. miR-34a-5p was consistently upregulated in heart and liver.	miR-34a can be used as a molecular marker of multi-system involvement.	Potential therapeutic targets	(105)
The 15 Most Relevant miRNAs (for details, please refer to the original text)	Cerebrospinal Fluid	Unknown	Seven mirnas were downregulated after treatment*.	miRNA changes reflect neurogenesis, neuronal differentiation and growth.	Monitoring of treatment response; Monitor disease progression; Potential therapeutic targets	(106)
42 Differential miRNAs (for details, please refer to the original text)	Human peripheral blood	Unknown	Fourteen mirnas were up-regulated and 28 mirnas were down-regulated between SMA patients and controls. 2 in the treatment of a significant rise in * 2 months; miR-423-3p and miR-142-5p were increased at 6 months.	The baseline levels of miR-107 and miR-142-5p were significantly correlated with treatment response. The levels of miR-142-5p and miR-378a-3p at 2 months after treatment predicted the treatment effect at 6 months.	Predicting treatment response; Treatment response was monitored	(107)

DMD, Duchenne Muscular Dystrophy; HINE-2, Hammersmith Infant Neurological Examination, Section 2; HFMSE, Hammersmith Functional Motor Scale Expanded; NMJ, Neuromuscular Junction; * Refers to nusinersen treatment.

At present, studies on the role of lncrnas in the pathological process of SMA are relatively limited. Existing evidence shows that lncrNA-mediated regulatory network disorders may be involved in the pathogenesis of motor neuron disease (MND) (40). Therefore, we hope that in the future, more scholars will further explore the expression characteristics, functions and regulatory mechanisms of lncrnas in SMA.

Studies in the field of circRNAs have found that circ4-2b-3 is consistently highly expressed in serum exosomes of some super-responders among patients with SMA type 1. Its expression level is closely correlated with the degree of improvement in motor function (≥7-point elevation in the CHOP INTEND score) after nusinersen treatment (41). This suggests that circ4-2b-3 may be the first circRNA biomarker for assessing response to nusinersen treatment in patients with SMA type 1.

### 2.4 Neurodegeneration

#### 2.4.1 Neurofilaments

Neurofilaments (NFs) are neuron-specific intermediate filaments and are classified into light chain (NF-L), medium chain (NF-M), heavy chain (NF-H), α-internexin, and peripherin (42). The expression levels of NF proteins in the CSF and peripheral blood are significantly correlated with the severity of axonal injury in inflammatory, degenerative, traumatic, and vascular neurological diseases (43). The diagnostic and prognostic roles of NF proteins in ALS are well-documented (44–46), and their value in SMA has been progressively confirmed (47).

Compared to healthy controls, plasma baseline pNfL levels were significantly higher in children with SMA type 2 or 3 (48). Adult CSF and serum NF-L levels were also significantly higher in patients with SMA, especially in those with SMA type 3 and “sitting patients” (49). Baseline CSF phosphorylated NF-H (pNF-H) levels were the highest in pediatric patients with SMA type 1; after nusinersen treatment, CSF pNF-H levels decreased significantly in all patients and remained elevated in all children. Moreover, CSF pNF-H levels decreased significantly and remained stable in all patients, and plasma pNF-H levels decreased significantly 2 months after treatment of patients with SMA type 1 or 2, but no significant differences were observed in patients with SMA type 3 (50). During long-term nusinersen treatment, NF-L concentrations were reduced (51). In addition, some studies have suggested that serum NF-L levels were significantly correlated with CSF NF-L levels, supporting the idea that these serum levels can be used as a surrogate marker for CSF levels in adult patients with SMA (49). Others found no association between sNF-L and cNF-L, but CSF and serum pNfH levels did correlate (52).

NF-L expression was significantly upregulated at the beginning of the treatment with nusinersen and then gradually returned to baseline levels (49). The use of onasemnogene abeparvovec for gene replacement therapy of serum NF-L levels showed paradoxical transient increases (53), possibly due to a transient immune response in the CNS or an inflammatory response resulting from the treatment. In addition, CSF NF-L and pNF-H levels may be useful biomarkers for the differential diagnosis of adult SMA and ALS (54).

Given the variety of causes of axonal injury, other motor neuron diseases should be strictly excluded before establishing neurofilament proteins (NFs) as specific biomarkers for spinal muscular atrophy (SMA). The application of NFs as a biomarker in SMA requires comprehensive consideration of factors such as patient age, clinical classification and disease stage. Previous studies have shown inconsistencies in the correlations between NF-L levels and motor function scores (48, 49, 55). NF-L levels are positively correlated with age (56), and serum NF-L levels are significantly positively correlated with disease duration (49). However, because of the intricate relationship between age and disease duration, more data are needed to explore whether disease duration affects NFs independently of age. Therefore, the use of serum NF levels as biomarkers of SMA still has some limitations.

#### 2.4.2 Tau

Similar to NF proteins, tau protein expression varies among the different clinical subtypes of SMA. In a mixed cohort study of adult patients with SMA type 3, tau proteins failed to show applicability as potential biomarkers during the loading period of nusinersen treatment because of the slow disease progression and its mild degree of neurodegeneration (57). However, a study from 2024 noted that the measurement of total tau protein concentration in the CSF was a reliable biomarker for monitoring the response to nusinersen therapy in patients with SMA types 1–3 (58). A more recent study found that tau levels in the CSF of nusinersen-treated patients with SMA type 1 were unchanged (59).

These conflicting findings prompted us to ask what key factors led to these discrepancies. We speculate that the reasons may involve several aspects. First, sample size is often a limitation, which renders small-scale studies susceptible to chance bias. Second, the variability of laboratory testing methods (e.g., sample processing procedures, analytical techniques, and adjudication criteria) may significantly interfere with the comparability of the data. Third, the heterogeneity of study populations is also a cause for concern, as as-yet-undiscovered biological differences in patients of different races, age groups, or clinical subtypes may exist. This suggests the need for larger, standardized, multicenter studies, as well as systematic analyses of potential confounders.

#### 2.4.3 Profilin

The profilin family of actin-binding proteins is essential in all organisms and plays a role in the regulation of the cytoskeletal structure, neuronal motility, and synaptic function (60). Among members of this family, profilin-1 is widely expressed throughout the body, whereas profilin-2 shows a CNS-specific expression pattern (61). Compared with profilin-1, SMN, through its proline-rich structural domain and profilin-2 binds more strongly (62, 63). However, a recent study (49) found that serum profilin-1 concentrations in patients with SMA decreased significantly during the first 2 months of nusinersen treatment; during the maintenance phase, profilin-1 decreased by 14.7% per month, eventually reaching levels similar to those of healthy controls after 26 months of treatment. The mechanism of action of the profilin family members in SMA has yet to be thoroughly investigated. The feasibility of using these markers for efficacy assessments must be verified in additional experiments.

### 2.5 Skeletal muscle

Creatine kinase (CK) and creatinine are involved in muscle energy metabolism, reflect muscle mass and muscle integrity, and are promising biomarkers for predicting and assessing responses to SMA treatment (64). Prior to nusinersen treatment, serum CK levels were strongly negatively correlated with disease severity scores, and the correlation of CK levels was greater than that of creatinine levels (65). During nusinersen treatment, adult patients with SMA had decreased CK levels and stable or slightly increased creatinine levels (65–67). CK is more suitable for assessing acute muscle injury or short-term response to treatment, whereas creatinine is more suitable for monitoring long-term trends in muscle atrophy. In addition, elevated urinary creatinine levels in patients with SMA type 1 correlate with improved motor function and may serve as a potential biomarker of treatment response (68). In addition, it has been found that myostatin expression is significantly decreased and follistatin expression is increased in muscle tissue in mouse and human SMA models. The low expression of myostatin in muscle tissue is related to the severity of the disease, may reflect denervated atrophy, or can be used as a marker of disease progression (69).

### 2.6 Inflammation and metabolism

Neuroinflammation is an emerging clinical feature in patients with SMA (70). The reason for these observed phenomena might be that elevated expression of pro-inflammatory cytokines in the spinal cord stimulates the proliferation of astrocytes and microglia of the pro-inflammatory M1 phenotype while activating the complement system (71). Some pro-inflammatory pathways (e.g., those involving CCL5 and TLR2) can exacerbate neuroinflammation by recruiting peripheral monocytes to the CNS (72).

Although the mainstream view in the past is that SMA pathological changes are closely related to neurological changes, evidence suggests that it may be a multisystem disease with pathological changes involving peripheral organs such as the heart, lungs, pancreas, and liver (73, 74). The liver is a key metabolic organ, and the loss of endogenous SMN protein in the liver can lead to hepatic injury and the replacement of hepatic tissue with fibrofatty tissue, causing systemic metabolic dysfunction (75). However, patients with SMA type 3 may have abnormal muscle-adipose tissue interactions (76), suggesting that muscle fat replacement may be related to insulin resistance and metabolic disorders in some patients with SMA.

Metabolic dysfunction in patients with SMA is characterized by significantly elevated levels of cholesteryl esters, decreased levels of free cholesterol, decreased phospholipid levels, increased lysophospholipid levels, abnormal high-density lipoprotein function, and increased levels of tryptophan and its metabolites (e.g., indoleacrylic acid) (77). Another study observed a significant negative correlation between glucose and lactate concentrations in the CSF and clinical improvement (Hammersmith Functional Motor Scale score) in adult patients with SMA type 2 or 3 (78). Moreover, serum copper concentrations in SMA are positively correlated with fat accumulation, and high copper may promote muscle degradation through oxidative stress or interfere with mitochondrial function, leading to muscle loss (79). These features may become biomarkers for disease diagnosis, staging, and therapeutic monitoring. Hepatic steatosis, dyslipidemia, abnormal iron metabolism, pancreatic endocrine imbalance, and other metabolic abnormalities improved after the correction of SMN protein levels in the liver (80).

Inflammatory and metabolic processes cannot be separated from cytokines, and researchers have screened some cytokines that are mainly related to SMA inflammation and have specific pathological significance (see Table 2 for details). Compared with CSF testing, peripheral blood cytokine analysis has advantages in convenience and economy. However, whether peripheral blood indicators can replace the cytokine changes in CFS is still controversial. In addition, inflammatory activity in the body is universal and can be triggered by various triggers, even daily physiological fluctuations (e.g., changes in diet and exercise). Therefore, despite the relative convenience of cytokine detection, the interference of background inflammation has limited its application as a biomarker for SMA. As for metabolic-related cytokines, since metabolic disorders are mainly secondary pathological changes in SMA, the authors believe that such cytokines should be used as secondary biomarkers in combination with other biomarkers and included in the multidimensional evaluation system of SMA.

**Table 2:** Characteristics and application analysis table of SMA inflammation-related cytokines.

Cytokines	Sample source	Expression pattern	Clinical association	Application	Reference
IL-23	Human peripheral blood; Human cerebrospinal fluid	It was elevated in serum and cerebrospinal fluid. After 6 months of treatment*, IL-23 levels were significantly reduced in the serum of pediatric patients, with no significant difference in CSF levels.	Pediatric patients with lower baseline serum IL-23 levels had better clinical outcomes after treatment*.	Prediction of treatment response	(108)
IL-10	Human peripheral blood; Human cerebrospinal fluid	It was elevated in serum and cerebrospinal fluid. After 6 months of treatment *, there were no significant changes in serum and CSF.	After 6 months of treatment*, higher levels of IL-10 in pediatric serum were positively associated with better HFMSE scores.	Treatment response monitoring	(108)
	Human cerebrospinal fluid	IL-10 did not show a significant level change during * 6 months of treatment.	Patients with higher baseline IL-10 levels had more significant improvement in motor function after treatment.	Prediction of treatment response	(109)
IFN-γ, IL-17A, and IL-22	Human peripheral blood; Human cerebrospinal fluid	It was elevated in serum cerebrospinal fluid.	This suggests activation of the Th1/Th17 immune pathway.	Potential therapeutic targets	(108)
Eotaxin, MIP-1β	Human cerebrospinal fluid	SMA1 patients were significantly reduced after 6 months of treatment *.	It is positively correlated with motor function improvement.	Treatment response monitoring	(109)
MCP-1, MDC, IL-8	Human cerebrospinal fluid	Treatment * initially increased transiently and returned to baseline after 6 months.	It may reflect an acute immune response (stimulated by drug injection).	Treatment response monitoring	(109)
CCL5, AMIGO1	Human peripheral blood	CCL5 was elevated in type 2 and 3 SMA and non-responders to treatment *. AMIGO1 was reduced in type 2 SMA and treatment * non-responders.	May indicate treatment * resistance.	Treatment response monitoring	(72)
CHIT1	Human peripheral blood; Human cerebrospinal fluid	It was significantly elevated during treatment *.	There was no significant correlation with clinical outcome and may reflect the inflammatory response induced by treatment.	Monitor the inflammatory response related to treatment	(52)
YKL-40	Human peripheral blood; Human cerebrospinal fluid	Levels decreased in CSF during the treatment phase*.	It was associated with an improvement in RULM scores during treatment*.	Treatment * Response monitoring	(52)

HFMSE, Hammersmith Functional Motor Scale Expanded; RULM, revised upper limb module; *refers to nusinersen treatment.

### 2.7 Other

Nusinersen can effectively increase the level of SMN protein in the central nervous system (CNS) and significantly improve the symptoms of patients. However, there are large numbers of non-responders, and treatment delay severely reduces efficacy – once motor neurons have entered an advanced stage of degeneration, their damage cannot be reversed by restoring SMN expression (81). Thus, it is critical to distinguish early between patients who have a response to Nusinersen and those who do not.

LARGE1 is a glycosyltransferase involved in neuromuscular function, and its increase may be associated with motor neuron protection or pathological processes. LARGE1 can be used as a potential biomarker for Nusinersen treatment response, especially in adult patients (82). LARGE1 protein was significantly increased in the CSF of adult SMA patients, especially in those who responded to nusinersen treatment. Serum LARGE1 levels were low at baseline in adult patients, increased significantly after treatment, and responders had significantly higher serum LARGE1 levels than non-responders. The level of LARGE1 in the CSF of pediatric SMA patients is already high at baseline and further increases in responders and decreases in non-responders after treatment, which can be used to monitor the response to treatment. However, there was no significant change in serum LARGE1 levels in pediatric patients.

However, we should not take treatment responders lightly. Clinical use of Nusinersen and Onasemnogene Abeparvovec (Zolgensma) has been associated with reported adverse events (83–85). The adverse reactions of Nusinersen involved 27 systemic organ categories, among which renal dysfunction, intrathecal injection complications and metabolic/psychiatric reactions were the potential risks that had not been fully appreciated. The adverse reactions of Onasemnogene Abeparvovec mainly included hepatotoxicity, hematological abnormalities, renal dysfunction, systemic reactions (fever, vomiting) and respiratory tract infection. For renal function, urine protein/creatinine ratio is more valuable than urine protein alone, and it is a more reliable index for evaluating renal function (68, 86).

These findings suggest that the implementation of individualized monitoring and treatment strategies is critical in the clinical management of SMA. Specifically, mechanisms for early identification of patients who have a poor response to treatment need to be established so that treatment can be adjusted in time to prevent disease progression to advanced stages and irreversible neuromuscular damage. At the same time, responders should be continuously monitored while receiving treatment, especially for potential adverse events that are not fully described in the drug labeling. This management strategy, based on stratification of treatment response, was designed to maximize clinical benefit and minimize associated risks.

## 3 Physiological biomarkers

In SMA, reduced levels of SMN protein trigger abnormal function and apoptosis of α-motor neurons in the spinal cord and in brainstem regions, which in turn progressively reduces the number of muscle fibers and decreases strength in trunk muscles including respiratory-related muscles, limb muscles, and swallowing-associated muscles (regulates by the medulla oblongata region).

### 3.1 Functionality

Maximal mouth opening (MMO) measurement is a part of the assessment of mandibular function and is a safe and easy-to-use method suitable for repeated testing. Due to medullary dysfunction, patients with SMA often have reduced MMO (87). In children aged 0–24 months, reduced MMO may be an early indicator of medullary functional involvement in SMA, particularly in children with two copies of the SMN2 gene (88). In adults, medullary functions, such as mandibular mobility and active MMO, decreased significantly over 4 years in patients with SMA type 2, but not in those with SMA type 3 (89).

Traditionally, SMA has not been thought to be directly related to primary heart disease. However, several studies have shown that arrhythmias and congenital cardiac structural abnormalities are not uncommon in SMA patients (90). Subclinical ventricular dysfunction is common in children with SMA (91). Notably, improvement in left ventricular (LV) function in children with SMA can be observed by echocardiographic monitoring after short-term Nusinersen treatment (92).

Perceived physical fatigability has been identified as the physiological factor most relevant to SMA pathophysiology. An assessment tailored to SMA patients, called SMA EFFORT, makes perceived physical fatigability ratings more objective, standardized, and better at assessing the impact of treatment on the physical health across the entire SMA spectrum (93).

### 3.2 Electrophysiology

The Hoffmann reflex (H-reflex), a spinal reflex evoked by electrophysiological methods, is primarily used to evaluate the excitability of spinal motor neurons and sensory nerve pathways. The H-reflex amplitude was reduced in mouse models of SMA and patients with SMA type 3 (26); after treatment, H-reflex function improved, along with significant improvements in motor function and fatigue in patients. H-reflex parameters can be used as quantitative biomarkers to track disease progression in real time and monitor treatment responses.

Important advances have recently been made in the electrophysiological assessment of the SMA by using two novel techniques, MScanFit motor unit number evaluation (MUNE) and disaggregated electromyography (dEMG), demonstrating their unique clinical applications (94). MScanFit focuses on the number of motor units and degree of reinnervation, whereas dEMG provides an in-depth analysis of the electrical discharge. dEMG complement each other and provide a multidimensional quantitative tool for the assessment of individualized therapeutic responses and the development of novel intervention strategies. However, longitudinal studies are needed to further validate its dynamic sensitivity in therapeutic monitoring.

## 4 Imaging biomarkers

Muscle fat replacement is common and often associated with various neuromuscular diseases (95). SMA can present with diffuse muscle fat replacement and has a complex pattern of muscle involvement: early onset of muscle fat replacement in the limbs, a strong correlation of fat content within the gastrocnemius muscle with disease severity and overall function, and relatively mild muscle fat replacement in medullary innervated muscles (e.g., masticatory and intrapterygoid). Significant fat accumulation has been observed in only a few patients (96). In seven nusinersen-treated children with SMA type 2 or 3, longitudinal MRI showed that an elevated intramuscular fat fraction of the thigh was significantly associated with decreased motor function (97). Meanwhile, patients with baseline IMFF < 20% had improvement in motor function (an increase in HFMSE score) after 4 years of treatment, whereas those with IMFF > 20% had a decline in function, suggesting that baseline IMFF can be used as a predictive marker of nusinersen response. Quantitative MRI study of eight children further found that although the thigh muscle fat fraction continued to increase after treatment, diffusion tensor imaging (DTI) showed that muscle microstructure may tend to be normalized, suggesting that IMFF can reflect the effect of drug intervention (98).

Muscle FF, as a biomarker, is of great value in SMA. FF can quantitatively provide objective muscle structure data through MRI, which can more accurately evaluate the patient’s condition and make up for the subjective limitations of traditional functional scores (such as HFMSE), especially for children and patients with severe movement disorders. FF can detect small changes in muscle at an early stage before complete factorization of muscle, which is more sensitive than clinical score. The baseline level and dynamic changes of muscle FF can be used as a long-term efficacy monitoring tool to help evaluate disease progression and treatment response.

## 5 Conclusion

**Figure 1:**
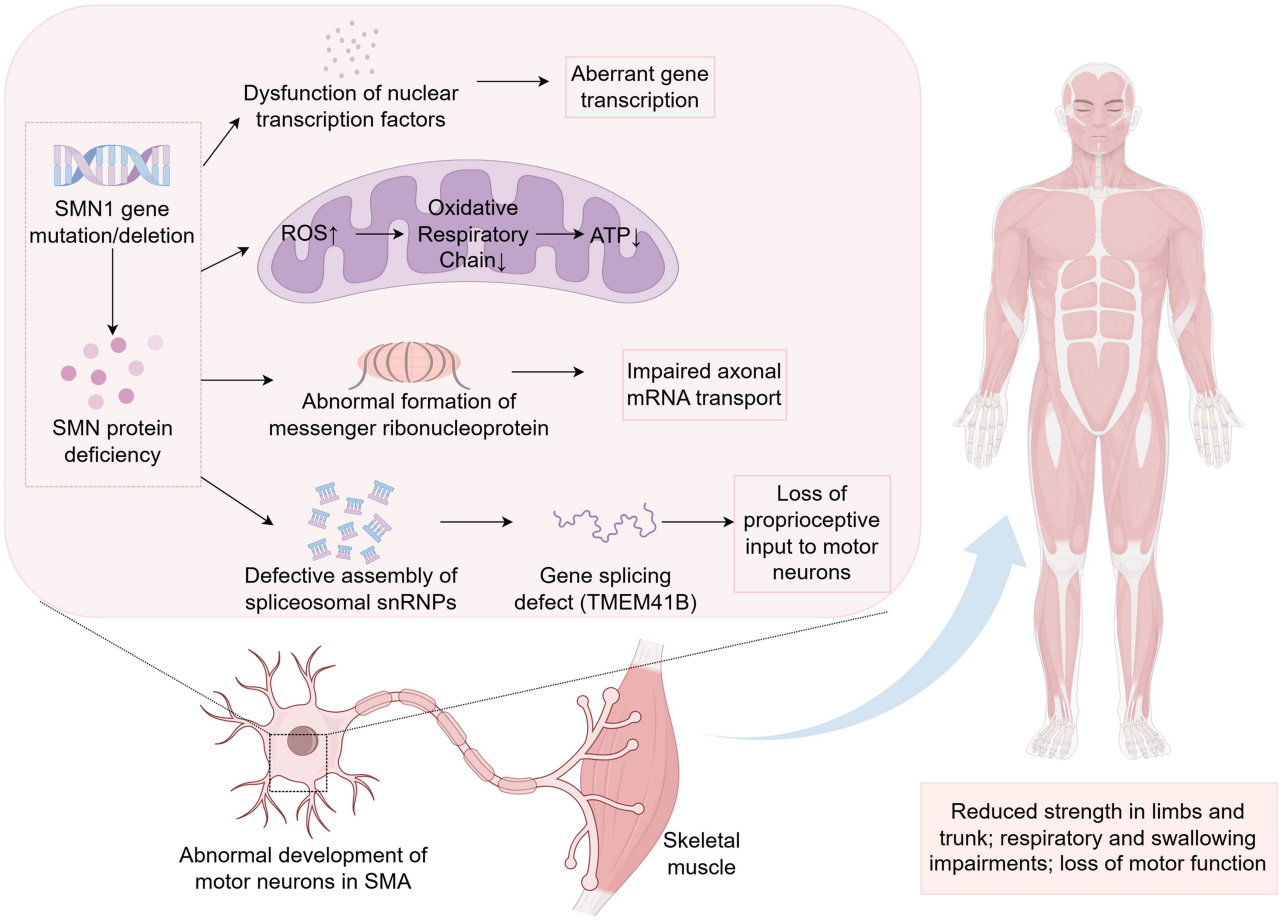
Pathogenesis of SMA (by Figdraw).

SMA is a rare disease, but the emergence of effective therapies has significantly prolonged the survival of patients. More specific biomarkers for diagnosis, treatment and prognosis are needed to help SMA patients achieve a better quality of life. This article systematically reviews the pathogenesis of SMA (Figure 1) and the biomarkers associated with SMA found in recent years (see Table 3 for details), focusing on molecular biomarkers. Among them, SMN protein level directly determines the survival ability of motor neurons and is still the core molecular marker of SMA. After the exclusion of other differential diagnoses and full consideration of confounding factors, NFs is still the most classical pathological index, which can reflect the state of nerve injury. Despite the increasing value of emerging molecular markers, their specificity and applicability still need to be further verified. In conclusion, the value of single indicator as a biomarker of SMA is limited, and the integration of multi-omics data analysis can obtain better evaluation effect.

**Table 3:** Summary of SMA-related biomarkers.

Biomarker	Reference
SMN protein	(15, 23–25)
Hsc70-4 and HspA8	(28, 29)
PLS3 and CHP1	(30–32)
TSP4	(35, 36)
miRNA	(71, 99–107)
circRNA	(41)
Neurofilament	(47–56)
Tau	(57–59)
Profilin	(49, 61–63)
Skeletal muscle (CK and Crn)	(64–66, 68, 69)
Metabolism-related cytokines	(77–80)
Inflammation-related cytokines	(52, 72, 108, 109)
LARGE1	(82)
Urine protein/creatinine ratio	(68, 86)
Maximum mouth opening	(88, 89)
Ventricular function	(90–92)
Degree of physical fatigue	(93)
Hoffmann reflex	(26)
MScanFit motor unit number estimation and decomposition electromyography	(94)
Intramuscular fat fraction	(96–98)

However, existing studies are generally limited by insufficient sample sizes, which not only lead to conflicting results of the same biomarker, but also weaken its association with clinical outcomes. In addition, SMA patient populations have significant heterogeneity (e.g., subtype, age at onset, baseline motor function), which, if adjusted for in analyses, may mask the true association of markers with clinical outcomes. But statistical irrelevance does not mean that these markers are not biologically meaningless. Potential reasons for the missing association may include the following: the index is not related to the core pathological mechanism; Study design flaws. The signaling pathways interfere with each other. In addition, negative results may also suggest that the role of this indicator in SMA is independent of the motor function pathway, and the research perspective needs to be repositioned. In future studies, we look forward to more reliable data to clarify the application of biomarkers in SMA and ultimately benefit SMA patients.
